# Vesicular Stomatitis Virus: From Agricultural Pathogen to Vaccine Vector

**DOI:** 10.3390/pathogens10091092

**Published:** 2021-08-27

**Authors:** Guodong Liu, Wenguang Cao, Abdjeleel Salawudeen, Wenjun Zhu, Karla Emeterio, David Safronetz, Logan Banadyga

**Affiliations:** 1 Special Pathogens Program, National Microbiology Laboratory, Public Health Agency of Canada, Winnipeg, MB R3E 3R2, Canada; guodong.liu@phac-aspc.gc.ca (G.L.); wenguang.cao@phac-aspc.gc.ca (W.C.); karla.emeterio@phac-aspc.gc.ca (K.E.); 2 Department of Medical Microbiology and Infectious Diseases, University of Manitoba, Winnipeg, MB R3E 0J9, Canada; salawuda@myumanitoba.ca; 3 Canadian Food Inspection Agency, National Centre for Foreign Animal Disease, Winnipeg, MB R3E 3M4, Canada; wenjun.zhu@inspection.gc.ca

**Keywords:** vesicular stomatitis virus, VSV, vaccine, countermeasure, Ebola virus, VSV-EBOV, reverse genetics, medical countermeasure

## Abstract

Vesicular stomatitis virus (VSV), which belongs to the *Vesiculovirus* genus of the family *Rhabdoviridae*, is a well studied livestock pathogen and prototypic non-segmented, negative-sense RNA virus. Although VSV is responsible for causing economically significant outbreaks of vesicular stomatitis in cattle, horses, and swine, the virus also represents a valuable research tool for molecular biologists and virologists. Indeed, the establishment of a reverse genetics system for the recovery of infectious VSV from cDNA transformed the utility of this virus and paved the way for its use as a vaccine vector. A highly effective VSV-based vaccine against Ebola virus recently received clinical approval, and many other VSV-based vaccines have been developed, particularly for high-consequence viruses. This review seeks to provide a holistic but concise overview of VSV, covering the virus’s ascension from perennial agricultural scourge to promising medical countermeasure, with a particular focus on vaccines.

## 1. Vesicular Stomatitis Virus

### 1.1. The Virus and Its Replication

The *Vesiculovirus* genus comprises a group of morphologically and genetically related viruses that infect mammals, birds, and reptiles [1]. Within this genus, the term “vesicular stomatitis virus” encompasses a number of related viruses belonging to a common serogroup, which is divided into the New Jersey and Indiana serotypes. The New Jersey serotype contains vesicular stomatitis New Jersey virus (VSNJV), while the Indiana serotype is further sub-divided into four distinct serological complexes. Indiana 1 contains vesicular stomatitis Indiana virus (VSIV), Indiana 2 contains Cocal virus (COCV), Indiana 3 contains vesicular stomatitis Alagoas virus (VSAV), and Indiana 4 contains Morreton virus (MORV). All of these VSVs, with the possible exception of MORV, are responsible for causing vesicular stomatitis disease in livestock animals in the Americas (described below), and VSIV is most commonly recognized as the type species for the genus *Vesiculovirus*. The abbreviation “VSV” will be used to refer, in general, to members of the VSV serogroup, with specific viruses identified as necessary. Notably, a number of other vesiculoviruses, such as Piry virus from South America, Chandipura virus from South Asia, and Isfahan virus from Western Asia, also cause disease in mammals but will not be considered in this review.

**Figure 1:**
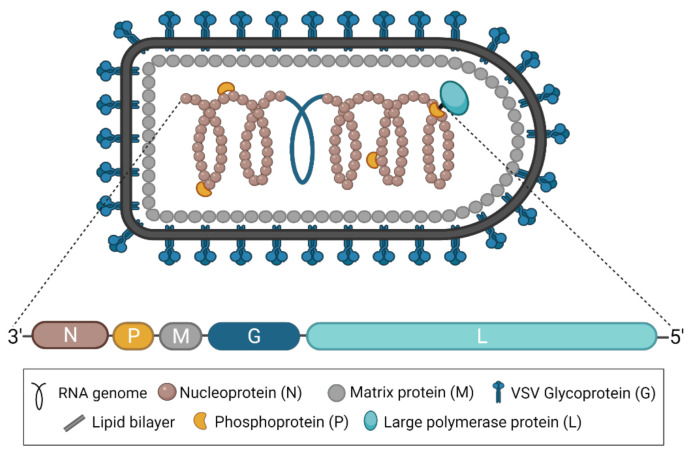
The structure of VSV and its genome. A diagram of the characteristically bullet-shaped virion is shown above a representation of the VSV genome. The negative-sense, single-stranded RNA genome (depicted in blue within the virion) is completely encapsidated by the nucleoprotein (N), which, together with the phosphoprotein (P) and the RNA-dependent RNA polymerase (L), forms the nucleocapsid or ribonucleoprotein complex. The matrix protein (M) condenses the nucleocapsid and drives virion budding. The glycoprotein (G) studs the surface of the virion and exists in trimeric complexes. This figure was created with BioRender.com.

As part of the family *Rhabdoviridae* and the order *Mononegavirales*, all vesiculoviruses possess a single-stranded, negative-sense RNA genome of approximately 11 kb [2]. In the case of VSV, this genome encodes five structural proteins: the nucleoprotein (N), the phosphoprotein (P), the matrix protein (M), the glycoprotein (G), and the RNA-dependent RNA polymerase (L) (Figure 1) [3]. VSNJV and VSIV both encode two additional non-structural proteins, known as C and C’, initiating from downstream start codons in the P gene [4,5]. Each gene contains conserved transcription start and end signals, and all genes are separated from each other by an intergenic region of two nucleotides [3]. The genome itself is flanked by a 3′ leader and 5′ trailer region, which contain transcription and replication promoters in addition to N protein encapsidation signals [3]. Together, the five structural proteins produce the bullet-shaped virion characteristic of rhabdoviruses [6], and the structures of each protein have been solved [7]. The viral genome is coated and protected by the N protein [8,9]. The P protein links the polymerase L [10] to the N-coated genome to form the nucleocapsid or ribonucleoprotein complex [11,12,13,14], which is condensed into a helical structure through interactions with the M protein [15,16]. The entire complex is wrapped in a cell membrane-derived envelope studded with G protein, which exists in trimeric complexes [17,18].

The VSV replication cycle has been extensively characterized and reviewed in detail elsewhere [19,20]. Briefly, the G protein mediates attachment to the low density lipoprotein receptor (LDLR), which, by virtue of its cellular ubiquity, accounts for the broad tropism of VSV [21,22]. Following attachment, the virion is thought to be endocytosed in a process that is dependent on both clathrin and actin [23]. As the virion subsequently travels through the endocytic pathway, a drop in pH triggers a conformational change in the G protein that exposes the hydrophobic fusion loop, which then inserts into the host endosomal membrane and facilitates its fusion with the viral envelope [23]. Fusion of the two membranes results in the release of the virion core into the cytoplasm of the host cell, where the M protein dissociates from the nucleocapsid to permit transcription and replication to occur [24].

VSV transcription and replication is driven by the viral polymerase complex, comprised of L and P, and the entire process is considered typical of single-stranded, negative-sense RNA viruses [19,20]. The first step is primary transcription of the initial viral genome, which generates a full complement of viral mRNAs, all of which bear 5′ methylated caps and 3′ poly-A tails. Importantly, the polymerase can only begin transcription at the 3′ end of the genome and move processively to the 5′ end, thereby, sequentially synthesizing N, P, M, G, and L mRNAs [25]. However, because the polymerase occasionally disengages at each intergenic region and then reinitiates transcription at the 3′ end of the genome, a transcriptional gradient is created, which results in reduced expression of a given viral protein the further its gene is located towards the 5′ end of the genome. Viral mRNAs are subsequently translated by host cellular machinery, and the accumulation of viral proteins, particularly N, is thought to trigger the polymerase’s switch from transcription to genome replication [26]. Replication is driven by a promoter in the 3′ leader of the negative-sense genome and produces a complementary, positive-sense RNA molecule known as the anti-genome, which is immediately encapsidated by N and subsequently serves as the template for the production of more genome. Newly synthesized and encapsidated genomes also serve as templates for the secondary transcription of viral genes, which, in combination with continued replication, represents the major amplification step of the replication cycle. Eventually, nucleocapsids are condensed via interactions with M protein, which also drive budding of nascent virions from the plasma membrane at sites that are enriched with G protein [27]. The entire VSV replication cycle, which ultimately kills the host cell, occurs relatively quickly and results in high titers of virus—two features that have made VSV an attractive tool in molecular biology research.

### 1.2. The Disease: Vesicular Stomatitis

Vesicular stomatitis is an acute disease of hoofed animals characterized by vesicular lesions primarily afflicting the oral mucosae and feet [20,28,29]. In cattle, lesions develop on the lips, tongue, gums, coronary bands, and/or teats, and, in pigs, vesicles develop on the snout, lips, coronary bands, and interdigital spaces. Likewise, in horses, lesions are present on the lips, tongue, gums, and palate, as well as the mammary glands and prepuce on mares and stallions, respectively. Disease is accompanied by a fever and may involve a period of viremia. Affected animals often develop anorexia, owing to the sores within and around the mouth, which can lead to weight loss and, in the case of dairy cows, decreased output. Animals typically recover completely in 2–3 weeks, although horses and pigs can develop lameness as a result of lesions around the hoof. Notably, in its early stages, vesicular stomatitis is clinically indistinguishable from the much more serious foot-and-mouth disease (caused by foot-and-mouth disease virus), making rapid diagnosis and reporting critical.

Vesicular stomatitis was first described following an outbreak in the United States in 1916 [30]; however, the disease was probably prevalent much earlier than that, with several descriptions of likely outbreaks stretching back to the early 1800s [29,31,32]. The disease is now understood to be enzootic to parts of the equatorial Americas, with a range extending from northern South America to southern Mexico, in addition to Ossabaw Island, off the coast of the U.S. state of Georgia. Epizootic outbreaks of disease occur seasonally, and stretch much farther south and north, through the central and southwestern U.S. up to Canada [31]. From one such outbreak, centered on Richmond, Indiana in 1925, VSIV was first isolated; similarly, VSNJV was isolated from another outbreak in New Jersey in 1926 [32]. The mechanisms of VSV maintenance and transmission in the wild are still incompletely understood, but evidence points toward the involvement of arthropod vectors, such as mosquitoes, blackflies, and sand flies [33]. Indeed, phlebotamine sand flies are known to contain virus in enzootic areas [34].

Humans are thought to contract VSV directly from infected animals [28] rather than from an arthropod vector, and seroprevalence can be high in enzootic areas [29]. Disease in humans is thought to most often be subclinical or mild, although more severe manifestations—including flu-like illness and the appearance of oral vesicular lesions—have been reported [35]. Moreover, although vesicular stomatitis is primarily considered to be a disease of livestock, neutralizing antibodies have been identified in a number of different wild animals, including deer, elk, coyotes, bears, skunks, squirrels, and rats, suggesting broad permissivity to VSV infection, if not susceptibility to disease [29].

## 2. VSV and Molecular Virology

### 2.1. VSV Reverse Genetics

As the prototype rhabdovirus, VSV has been widely used as a model system to study the replication and assembly of mononegaviruses. Moreover, the VSV genome has been engineered for use as a research tool, a vaccine platform, and an oncolytic vector [36]. Most of these applications require the use of the VSV reverse genetics system that allows for direct manipulation of the VSV genome using recombinant DNA techniques.

There are several challenges associated with generating infectious virus from a cDNA clone of the VSV genome, as is the case for all negative-sense RNA viruses. Unlike positive-sense RNA viruses, for which the genome is inherently infectious, negative-sense viruses, such as VSV, must first undergo transcription to produce the viral proteins required for subsequent rounds of transcription and genome replication. In addition, both negative-sense genomes and positive-sense antigenomes must be encapsidated by the nucleoprotein in order to be recognized by the viral RNA polymerase complex as templates for replication and transcription. Thus, in the case of VSV, the viral proteins N, P, and L—which, along with the RNA genome, make up the ribonucleoprotein (RNP) complex—need to be provided in trans.

A breakthrough was made in 1994 when the infectious rabies virus, a close relative of VSV in the *Rhabdovirus* family, was generated entirely from cloned cDNA [37]. Less than a year later, VSV itself was recovered from a full-length cDNA clone by two labs independently [38,39]. The secret of successful recovery of VSV and the rabies virus largely relied on the use of plasmids encoding an anti-genomic instead of genomic RNA analogue, with the inclusion of the hepatitis delta virus (HDV)-derived ribozyme sequence to produce accurate 3′ ends. N, P, and L proteins were provided in trans from so-called “helper” plasmids, and expression from all plasmids was driven by a T7 promoter. When all the plasmids were transfected into BHK21 cells co-infected with recombinant vaccinia virus expressing the T7 RNA polymerase, the infectious virus was successfully rescued.

Using similar techniques, reverse genetics systems have since been established for numerous other negative-sense RNA viruses from the *Paramyxoviridae*, *Filoviridae*, and *Bornaviridae* families, and the process has been progressively optimized over time [40]. In addition to precise processing of the 3′ ends of the initial round of anti-genomic RNA molecules, precision at the 5′ end is also critical. For this purpose, the hammer-head ribozyme sequence has been introduced at the 5′ end of the full-length viral cDNA to ensure precise cleavage prior to the leader sequence [41,42]. Moreover, the conditions under which the viral RNP proteins are provided have been made more feasible. Expression of these proteins can be achieved either transiently from co-transfected plasmids or via stably transfected cell lines used for rescue. Vaccinia virus-free reverse genetics systems have also been developed, by either expressing the T7 polymerase from a co-transfected plasmid or by replacing the T7 promoter with a cellular RNA polymerase II-driven promoter, such as the CMV promoter [43,44,45].

The establishment of plasmid-based VSV reverse genetics systems opened the possibility to manipulate the VSV genome, which greatly facilitated studies of cell tropism, replication cycle, and pathogenesis of VSV and related viruses. More importantly, these reverse genetics systems have provided the basis for the development of VSV as a widely used research tool, as well as a vaccine platform and oncolytic vector.

### 2.2. VSV as a Molecular Tool

VSV has several properties that make it a valuable tool for the study of molecular virology. First, the biology of VSV is well-studied. Since the recovery of infectious VSV from cDNA using reverse genetics, the virus has been extensively studied in various respects, including cell entry, virus-host receptor interaction, and replication/transcription. Second, VSV has a relatively small genome of about 11 kb that is easy to manipulate. In addition, the VSV genome has the capacity to stably accept relatively large (4–5 kb) gene insertions [46], and these genes can be expressed at high levels. Thirdly, VSV replicates quickly and to high titers in most mammalian and insect cell lines, which makes it easier to produce in large quantities and, thereby, facilitates the practical applications of this tool. Finally, VSV has the capability to express unrelated glycoproteins on the surface of the virion.

**Figure 2:**
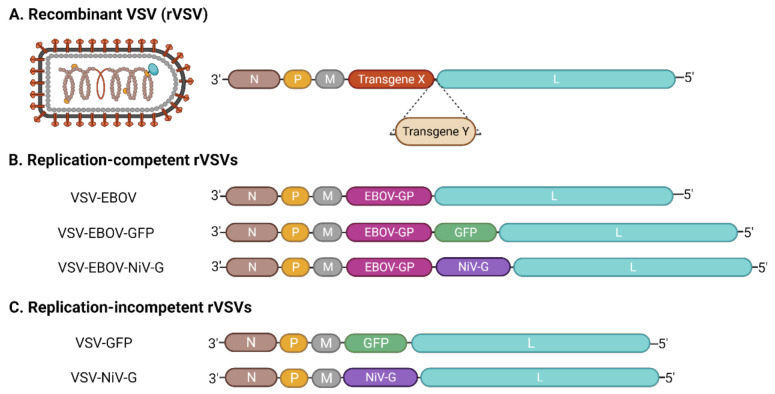
Recombinant VSVs. (**A**) Manipulation of the VSV genome results in recombinant VSVs (rVSVs) that can be used for a variety of purposes. Most rVSVs contain additional transgenes (i.e., transgene X or Y)—often in place of VSV G—that drive the expression of additional proteins, including other viral glycoproteins or reporter molecules. Insertion of one or more transgenes is flexible and can be accommodated at almost any position within the genome. (**B**) Replication-competent rVSVs result when the added transgene supports virus replication. For example, in the case of VSV-EBOV, the EBOV GP replaces VSV G but is still sufficient to facilitate the VSV replication cycle [49]. VSV-EBOV-GFP expresses GFP in addition to EBOV GP [50], while VSV-EBOV-NiV G expresses both EBOV GP and the NiV attachment glycoprotein [51]. (**C**) Replication-incompetent rVSVs result when the added transgene cannot support virus replication. For example, replacement of VSV G with GFP results in a virus that must be pseudotyped with a glycoprotein in order to be infectious [52]. Similarly, NiV G is unable to facilitate virus replication in the absence of VSV G [53]. This figure was created with BioRender.com.

Currently, there are two types of recombinant VSVs (rVSVs) that are widely used as tools in virology research: replication-incompetent rVSV and replication-competent rVSV (Figure 2). The replication-incompetent rVSVs typically contain a genome in which the VSV G gene has been deleted (VSVΔG) and replaced with a gene encoding a fluorescent or luminescent reporter. Meanwhile, during virus rescue, transient expression of a heterologous glycoprotein from a different virus results in the production of “pseudotyped” virions studded with the non-native glycoprotein. Since the genomes of replication-incompetent rVSVs do not contain a glycoprotein gene, the viruses are only capable of replicating for a single round, unless glycoprotein is supplied in trans. Conversely, replication-competent rVSVs have genomes in which the VSV G gene has been swapped for that of a heterologous glycoprotein, thus, producing a pseudotyped virus capable of multiple rounds of replication. In addition, these viruses can be engineered to encode a reporter gene to facilitate quantitative applications. Both types of rVSVs have been widely used in various applications for the study of different viruses [36,47]. Indeed, rVSVs are routinely used to study cell tropism, cell entry, and virus–host interactions of different viruses. They are also frequently used in high-throughput screens of antivirals/inhibitors that target cell entry—taking advantage of the reporters expressed from the virus genome. rVSVs can also be used to detect, evaluate, and titer neutralizing antibodies. Notably, both replication-competent and replication-incompetent rVSVs can be used safely in biosafety level 2 (BSL-2) laboratories, which has made them especially useful for studying the properties of many high-containment viruses that would otherwise be inaccessible to the majority of research laboratories around the world.

It is worth noting that, while pseudotyped rVSVs are common molecular tools, the converse use of VSV G to pseudotype other viral particles is also common [48]. Indeed, its wide-ranging tropism and good stability have made VSV G one of the most widely used viral glycoproteins to pseudotype lentiviruses. These VSV G-pseudotyped lentiviruses, in turn, have proven invaluable not only as a tool for experimental gene knockdown and overexpression but also as a vector for gene transduction and gene therapy [36].

## 3. VSV as a Vaccine Vector

Many of the properties that make VSV a valuable tool for molecular virology research—such as its ability to express heterologous glycoproteins and replicate to high titers—also make it an excellent vaccine platform. In addition, the apparent seroprevalence of VSV antibodies is generally low within the human population, so there is little pre-existing immunity to the vector, and because the VSV replication cycle takes place exclusively in the cytoplasm of host cells, there is no potential for viral sequences to integrate into the host genome. As a result, over the past several years, rVSV has emerged as a viable vaccine vector, and many rVSV-based vaccines have demonstrated safety and immunogenicity in multiple pre-clinical and clinical trials.

The history of VSV-based vaccine development is closely intertwined with research on high-consequence pathogens—especially Ebola virus (EBOV). The early promise and eventual success of a VSV-vectored EBOV vaccine has since led to the development and characterization of a number of other candidate vaccines against emerging and deadly viruses. Below, we have focused our discussion on the VSV-based EBOV vaccine, for which data abound. However, we also describe VSV-based vaccines that have been developed for many other high-consequence pathogens, including Marburg virus and other filoviruses, Lassa virus, henipaviruses, and coronaviruses.

### 3.1. Ebola Virus

#### 3.1.1. Pre-Clinical Development

VSV was first used in the study of filoviruses for the functional characterization of the EBOV glycoprotein (GP) [52,54,55]. Interest in using VSV as a vaccine vector against EBOV grew out of the success in generating a set of replication-competent rVSIVs expressing the glycoproteins of several high-consequence viruses, including EBOV [49]. An EBOV GP-pseudotyped rVSV, initially named VSV∆G/ZEBOVGP and now widely called VSV-EBOV or rVSV-ZEBOV, was generated by replacing the VSV G gene [56] with the GP gene of EBOV (variant Kikwit). An initial test of its ability to provide prophylactic protection against mouse-adapted EBOV (MA-EBOV) in mice was highly successful. VSV-EBOV itself did not cause disease in mice, yet it resulted in 100% survival of the animals after being challenged with a lethal dose of MA-EBOV following two doses of 2 × 10^4^ plaque-forming-units (PFU) of intraperitoneally delivered VSV-EBOV [49]. Similar results were observed in guinea pigs [49]. A subsequent study in cynomolgus macaques demonstrated that a single dose of 10^7^ PFU of VSV-EBOV via intramuscular injection caused no disease in the animals and fully protected them from EBOV challenge 28 days later [57]. This set of studies demonstrated that replication-competent rVSV is a safe viral vector and that VSV-EBOV is a promising vaccine candidate against EBOV.

VSV-EBOV has since undergone extensive pre-clinical studies to evaluate its efficacy under different vaccination strategies and viral challenge methods. It was further assessed in mice using decreasing vaccine doses, with as little as 2 PFU of VSV-EBOV showing full protection from challenge with a lethal dose of 10^3^ LD_50_ of MA-EBOV given 28 days post-vaccination [58]. Likewise, a higher single dose of 2 × 10^4^ PFU of the vaccine given intraperitoneally, intramuscularly, or intranasally provided full protection against up to 10^6^ LD_50_ of MA-EBOV. Immunization of cynomolgus macaques with 2 × 10^7^ PFU of VSV-EBOV via the oral or intranasal route also conferred complete protection against disease, as did immunization via the intramuscular route [59], suggesting the effectiveness of the vaccine is independent of administration routes.

VSV-EBOV was further evaluated for its ability to provide rapid protection soon after vaccination or challenge. The vaccine proved to be fully protective in mice when given 7 days before challenge with a lethal dose of MA-EBOV [58], and it also offered full protection when administered to mice only 24 h before virus challenge [60]. VSV-EBOV was also effective in guinea pigs, conferring survival rates of 66% when given 24 h before challenge, and 87% or 50% when given 1 h or 24 h, respectively, after challenge with a lethal dose of guinea pig-adapted EBOV (GPA-EBOV) [60]. In hamsters, VSV-EBOV conferred 100% survival when given as late as 1 day after MA-EBOV challenge [61]. Remarkably, in rhesus macaques, one dose of 2 × 10^7^ PFU of VSV-EBOV provided 50% protection when given 20 to 30 min after challenge with EBOV variant Kikwit [60]. In addition, the same dose of VSV-EBOV administered 24 h post-challenge protected 67% of rhesus macaques from EBOV variant Makona, the agent responsible for the 2013–2016 West African EBOV epidemic [62]. Together, these studies demonstrate that VSV-EBOV can induce rapid protection and be used as a post-exposure treatment.

The durability of the protection offered by VSV-EBOV is another important feature of the vaccine and was also assessed in rodents. A single dose of 2 × 10^4^ PFU of VSV-EBOV provided full protection from MA-EBOV in mice up to 9 months after vaccination, and 2 × 10^5^ PFU of the vaccine conferred full protection against GPA-EBOV in guinea pigs up to 18 months post-immunization [58,63]. The results indicated VSV-EBOV is able to provide long-term protection.

#### 3.1.2. Clinical Trials and Approval

The need for effective medical countermeasures against EBOV became extremely urgent during the 2013–2016 West African epidemic—the largest EBOV outbreak so far recorded. Since late 2014, many therapeutics and vaccines have been evaluated in clinical trials, with VSV-EBOV evaluated in over 10 clinical investigations covering all three stages of clinical trials. Recent publications have reviewed these clinical studies in detail [64,65], and thus, a brief summary of published studies and an update of more recent clinical trials are provided in this review.

A series of phase 1 or phase 1/2 clinical trials in adult participants, including many dose-escalation studies, were conducted in countries across North America, Europe, and Africa [66,67,68,69,70,71,72]. Generally, transient VSV-EBOV shedding lasting up to approximately 3 days was observed among the participants. Typical adverse events included injection-site pain, headache, fever, and myalgia, which are common for live virus vaccines and mostly mild and self-limiting. Notably, however, VSV-EBOV immunization has been associated with higher frequencies of arthritis, perhaps owing to immune cell targeting mediated by EBOV GP, as well as rare instances of cutaneous lymphocytic vasculitis [66,67]. Robust antibody responses against EBOV-GP, in addition to neutralizing antibody titers, were observed in most vaccinees. A randomized, non-controlled phase 1 trial, comprising adults as well as adolescents aged 13–17 years and children aged 6–12 years, showed that VSV-EBOV caused no serious adverse reactions, though higher levels of vaccine replication and shedding of virus in saliva and urine was observed in the non-adult groups [73]. High levels of EBOV GP-specific antibody titers were detected in the participants 28 days post-vaccination, and these titers persisted or increased up to 3 months. Neutralizing titers were detected in over 50% of the participants, with children showing significantly higher titers of neutralizing antibodies compared to the adults and adolescents. Analysis of blood samples collected from 30 healthy participants of two of the clinical studies [67,70] detected antibody responses against the VSV matrix protein and CD8+ T cell responses against the VSV nucleoprotein, which were both undetectable before vaccination [74], suggesting pre-existing immunity against VSV-EBOV is unlikely in humans. Altogether, these clinical studies demonstrated that VSV-EBOV is generally safe and highly immunogenic. These studies also identified an optimal dose of 2 × 10^7^ PFU, which has since been widely used in vaccination.

Following the success of early-stage clinical studies, VSV-EBOV was advanced into phase 3 trials to further evaluate its protective efficacy. The first trial, Ebola ça Suffit (meaning “Ebola this is enough”), which started in Guinea in 2015 and was later expanded to Sierra Leone, employed a unique design called ring vaccination. Based on this method, a cluster (or “ring”) of contacts and contacts-of-contacts of EBOV-positive patients was immunized with VSV-EBOV (2 × 10^7^ PFU) either immediately or 21 days after case confirmation. The final results revealed that no cases of Ebola virus disease occurred at least 10 days after vaccination among the 5837 vaccinees who received the vaccine immediately, whereas 23 cases were identified among those participants who received the vaccine later or not at all [75]. The results showed an estimated 100% efficacy and demonstrated that VSV-EBOV is highly effective in providing rapid protection amid an outbreak. In a randomized phase 2/3 clinical study known as the Sierra Leone Trial to Introduce a Vaccine against Ebola (STRIVE), there were no EVD cases or serious vaccination-related adverse events identified among the participants [76]. Another randomized phase 3 trial conducted in North America and Europe evaluated the safety and immunogenicity of VSV-EBOV at a dose of 2 × 10^7^ PFU and a high dose of 1 × 10^8^ PFU [77]. The vaccine was generally well-tolerated among the groups and caused no serious adverse events.

Taken together, VSV-EBOV has proven to be a highly effective vaccine candidate with multiple clinical trials demonstrating efficacy across diverse geographic and demographic settings. Indeed, during the more recent outbreaks in the Democratic Republic of the Congo, VSV-EBOV was swiftly implemented as the first choice of preventive countermeasure and played an important role in controlling disease spread [78,79]. In 2019, as a result of extensive pre-clinical and clinical evaluation demonstrating remarkable safety and efficacy, the U.S. Food and Drug Administration licensed VSV-EBOV (also known by its brand name, Ervebo) for use in humans against EBOV infection [80].

#### 3.1.3. Mechanisms of Protection

With tremendous effort devoted to evaluating the efficacy of VSV-EBOV, a lot of interest has also been focused on understanding how this vaccine provides protective immunity. Early studies showed that humoral immunity induced by VSV-EBOV likely plays a main role in the protection against EBOV. Passive transfer of serum from mice immunized with VSV-EBOV to unvaccinated mice protected 80% of the animals from MA-EBOV infection, an effect not seen by transferring naïve serum or immune serum from mice inoculated with wild type rVSV [58]. In mice that were immunized with VSV-EBOV, GP-specific IgM and IgG responses were detected by ELISA after one week, with a nearly 20-fold decrease in IgM levels and a five-fold increase in IgG2a levels by day 28 post-immunization. Intriguingly, neutralizing activity was inconclusive in these mice. Similarly, increasing EBOV GP-specific IgG titers were detected in cynomolgus macaques following immunization of VSV-EBOV, while neutralizing antibody titers were absent before EBOV challenge (but present afterwards) [57]. Several clinical trials have since provided the opportunity to characterize vaccine-induced anti-GP immune responses in humans. One study profiled the antibody response elicited by VSV-EBOV in participants of a phase 1 clinical trial [81], demonstrating a diverse profile of GP-specific antibodies exhibiting strong binding and neutralization capabilities. Interestingly, this study also revealed high levels of IgM antibodies with neutralizing activity, suggesting that this antibody isotype may play an important role in vaccine-induced immunity against EBOV. Another study, focusing on the humoral response in a small subset of vaccinees, identified robust polyclonal but convergent antibody responses that, in each individual, resulted in highly potent neutralizing antibodies [82]. Overall, these studies demonstrate that an antibody response is essential to the immune protection conferred by VSV-EBOV.

Studies on the role of cellular immunity induced by VSV-EBOV have presented mixed results, although the contribution of CD4+ T cells to the humoral immune response is clear. In a study involving immunocompromised rhesus macaques infected with simian-human immunodeficiency virus (SHIV), VSV-EBOV vaccination was shown to protect four of six animals from death due to EBOV infection [83]. The two animals that succumbed to infection exhibited the lowest CD4+ T cell count, suggesting that these cells may play a role in the immune protection offered by VSV-EBOV [83]. This observation was corroborated by a more recent study that focused on the immunological mechanisms underlying VSV-EBOV-mediated protection [84]. Cynomolgus macaques that were depleted of CD4+ T cells at the time of VSV-EBOV vaccination did not mount an anti-GP IgG response and succumbed to infection. Conversely, however, animals that were depleted of CD4+ T cells at the time of EBOV infection, rather than at the time of vaccination, produced an EBOV-specific antibody response and survived. These results demonstrate that while CD4+ T cells do not play a direct role in VSV-EBOV-mediated protection, they do play an indirect role, presumably by supporting a robust B cell response. The subsequent discovery that levels of circulating follicular helper T cells (a subset of CD4+ T cells) positively correlate with anti-GP antibody titers in human VSV-EBOV vaccinees provides additional evidence for the indirect role of CD4+ T cells in the immunity elicited by VSV-EBOV [85]. Above all, these results further underscore the importance of an antibody response in conferring immunity against EBOV.

The importance of CD8+ T cell responses to VSV-EBOV-mediated immunity is less clear. Depletion of CD8+ T cells 25 days post-vaccination in mice [58] or 7 days prior to vaccination in cynomolgus macaques [84] had no impact on protection against EBOV, suggesting that CD8+ T cells do not play a significant role in VSV-EBOV-mediated protection. On the other hand, profiling of cellular immunity in vaccinees demonstrated activation of CD8+ T cells 7 days post-vaccination [70]. Although the response was low to moderate in magnitude and no significant expansion of polyfunctional CD8+ T cells was observed at day 56, some contribution to vaccine-induced immunity cannot be ruled out. Interestingly, analysis of CD8+ T cell immunity among survivors of the 2013–2016 EBOV epidemic identified the viral nucleoprotein as the most dominant target of CD8+ T cells responses, with GP identified as a minor target [86], suggesting GP may not be a strong stimulator of CD8+ T cell response after all. More work is required to clearly establish a role for the CD8+ T cell response in VSV-EBOV efficacy.

The ability of VSV-EBOV to confer some level of protection immediately before or after EBOV inoculation suggests that innate immune responses to the vaccine are also critical. NK cells have been implicated in protecting 50% of rhesus macaques inoculated with EBOV 20–30 min prior to treatment with VSV-EBOV [60], and this cell type has proven critical to the rapid protection offered by a virus-like particle-based vaccine in mice [87]. Furthermore, three days after immunization with VSV-EBOV, cynomolgus macaques showed increased levels of cytokines associated with NK cell and macrophage activation [88]. These results were corroborated by transcriptomic analyses, which revealed that the magnitude of the innate immune transcriptional response at day 3 post-vaccination correlated with viral burden and disease outcome [89]. Collectively, the cellular and molecular signatures associated with the activation of an innate immune response shortly after VSV-EBOV vaccination are suggestive of a generic role for innate immunity in providing rapid protection.

#### 3.1.4. Challenges

Although VSV-EBOV has proven to be highly efficacious in many pre-clinical and clinical studies, there have been some concerns about its effectiveness and safety throughout development. One question often raised against a viral vaccine vector is whether pre-existing immunity to the vector itself will re-shape the immune response against the vaccine and compromise its efficacy. It has previously been reported that pre-existing immunity against VSV is generally limited among humans [90], and removal of VSV G in most vaccine constructs is expected to further mitigate the impact of potential pre-existing immunity. Nevertheless, to evaluate the impact of anti-VSV immunity on the effectiveness of VSV-EBOV, cynomologus macaques that had survived infection with Lassa virus (LASV) following immunization with a VSV vaccine expressing the LASV glycoprotein (VSV-LASV) were immunized again with 10^7^ PFU of VSV-EBOV [91]. All animals developed a robust EBOV GP-specific antibody response, and all survived challenge with a lethal dose of EBOV, indicating that pre-existing immunity against VSV had no impact on the effectiveness of VSV-EBOV.

Because wild-type VSV is neurotropic and can cause neurological disorders in rodents and primates [92], the potential for neurovirulence of VSV-EBOV has been considered. Cynomologus macaques were inoculated with 10^7^ PFU of VSV-EBOV or VSV-MARV (discussed below) through intra-thalamic injection; however, the animals did not develop any neurological symptoms and exhibited only mild histologic changes in the brain [93]. In contrast, the control animals that received wild type VSV demonstrated severe neurological symptoms and profound histological lesions in the brain and spinal cord. Conversely, a recent study in neonatal mice demonstrated that VSV-EBOV was associated with neurological symptoms, virus replication in the eyes and brain, and death in inoculated animals [94]. Thus, while nonhuman primates do not appear susceptible to neurovirulence caused by VSV-EBOV, the results from neonatal mice suggest that vaccine may pose a risk to the very young, including human infants. Along similar lines, the tolerability of VSV-EBOV in immunocompromised individuals was tested in NOD-SCID mice [58] and immunodeficient rhesus macaques (infected with SHIV) [83]. Despite receiving very high doses of VSV-EBOV, neither the mice nor the macaques showed any signs of disease related to immunization. Moreover, four of the six macaques survived a lethal challenge of EBOV. These data suggest that VSV-EBOV is well tolerated and at least partially protective in individuals with compromised immune systems.

Finally, the potential of VSV-EBOV to cause spillover infection and disease in livestock animals has also been assessed. In an initial study, pigs inoculated with 10^6^ PFU of VSV-EBOV showed low levels of virus shedding and no obvious signs of disease [95], suggesting that spillover of the vaccine virus into animal populations is unlikely to pose a significant risk. A follow up study, using an even higher dose of VSV-EBOV (4 × 10^7^ PFU), did result in vesicular lesions on the snout and feet of inoculated pigs, but—importantly—the virus was not transmitted to naïve pigs in close contact [96]. Thus, it appears that VSV-EBOV is a safe and effective human vaccine that poses little threat to livestock animals.

### 3.2. Marburg Virus and Other Filoviruses

The development of rVSV-vectored vaccines against other filoviruses has paralleled the development of VSV-EBOV, although none has progressed as far. VSV-MARV, which encodes the Marburg virus (MARV) GP in place of VSV G, is analogous to VSV-EBOV and was initially generated at the same time [49]. Subsequently, it was shown that a single dose of 10^7^ PFU VSV-MARV (encoding GP from the Musoke variant) could completely protect cynomolgus macaques from lethal MARV (variant Musoke) infection 28 days post-vaccination [57]. Further, the same vaccine, administered at a dose of 2 × 10^7^ PFU, was able to provide full protection to cynomolgus macaques following challenge with the MARV variant Angola or the related Ravn virus [97], indicating that the vaccine confers protection against multiple different marburgviruses. Strikingly, the immunity provided by VSV-MARV is durable: cynomolgus macaques challenged with MARV 14 months after vaccination were completely protected and did not show any clinical signs of disease [98]. Similar to VSV-EBOV, VSV-MARV is also able to protect animals when administered post-exposure, although the efficacy may depend on the variant and dose of MARV used to challenge animals. VSV-MARV (expressing Musoke GP) given 20–30 min after inoculation with MARV (variant Musoke) was sufficient to protect 100% (5/5) rhesus macaques [99], and when vaccine administration was extended to 24 or 48 h post-inoculation, 83% (5/6) and 33% (2/6) animals were still protected [100]. VSV-MARV (expressing Angola GP) given within 20–30 min after inoculation with MARV (variant Angola) protected only 25% (1/4) of the animals; however, when lower doses of MARV were used, survival improved to as high as 75% (3/4) [101]. Similar to the VSV-EBOV vaccine, the production of GP-specific antibodies seems to be critical to protection [57], aided by appropriate innate immune signaling and a Th1 T-cell response [102,103].

The success of VSV-EBOV and VSV-MARV has been recapitulated with VSV-based vaccines against other human-pathogenic filoviruses, particularly Sudan virus (SUDV) and Bundibugyo virus (BDBV) [104,105]. Moreover, since GP-based rVSV vaccines against filoviruses offer limited cross-protection against heterologous viruses [57,104,106,107], a number of different approaches have been used to develop multivalent VSV vaccines. Blended, single-dose vaccines consisting of a mixture of different VSV vectors, each expressing a different GP, have proven successful in nonhuman primate models against all human-pathogenic filoviruses, as well as LASV [108,109,110]. Similarly, a single rVSV expressing three different filovirus GPs was able to protect guinea pigs from EBOV, MARV, and SUDV [111].

### 3.3. Lassa Virus

LASV, a member of the *Arenaviridae* family, is the etiologic agent of Lassa fever, a severe viral hemorrhagic fever that is common in West Africa. The VSV-based LASV vaccine, which expresses the LASV pre-glycoprotein complex (GPC) in place of VSV G, traces its origins to the same study that produced VSV-EBOV and VSV-MARV [49]. Although this vaccine, referred to here as VSV-LASV but also known as rVSV∆G/LASVGPC, has proven to be highly efficacious in animal models, its clinical development has lagged far behind that of VSV-EBOV.

In the first study to demonstrate the efficacy of VSV-LASV, four cynomolgus macaques were vaccinated intramuscularly with a single dose of 2 × 10^7^ PFU and were challenged with 1 × 10^4^ PFU of LASV (strain Josiah) 28 days later [112]. Blood samples collected prior to challenge demonstrated moderate-to-high levels of LASV-specific IgG antibodies, including low-titer neutralizing responses, in all animals. After virus challenge, none of the animals manifested clinical signs of disease, and biochemical and hematological values remained within normal or near-normal limits. Viremia was noted on day 7 post-infection, but it was gone by day 10. Importantly, CD8^+^ T cells were detected in three of the four vaccinated macaques, suggesting both cellular and humoral immunity may be involved in the protection afforded by VSV-LASV.

Unlike EBOV, LASV isolates display significant genetic variability, raising the question of whether the VSV-LASV vaccine could provide protection from a heterologous LASV strain. To address this, strain 13 guinea pigs were immunized with 10^6^ PFU of VSV-LASV (expressing G from the Josiah strain of LASV) and then challenged 28 days later with 1 × 10^4^ TCID_50_ of LASV (strain Josiah) or a variety of heterologous strains [113]. Not only did VSV-LASV protect animals from the Josiah strain, but it also protected them from Liberian (LASV Z-132), Malian (Soromba-R), and Nigerian (LASV Pinneo) strains, further demonstrating the efficacy and broad cross-reactivity of this vaccine. Indeed, immunization of cynomolgous macaques with 1 × 10^7^ PFU of VSV-LASV followed by challenge with a lethal dose of the Liberian LASV strain resulted in complete protection of the animals and no signs of disease, corroborating the guinea pig data as well as the initial nonhuman primate studies.

An important feature of any vaccine is the duration for which it provides immune protection, and VSV-LASV has been shown to elicit an immune response that persists for at least 12 months [114]. A dose of 10^6^ PFU of VSV-LASV protected 87.5% and 77% of Hartley guinea pigs following challenge with guinea pig-adapted LASV at 189 and 355 days, respectively. While most of the vaccinated guinea pigs had peak LASV-specific IgG titers by 51 days post-vaccination, the protected animals exhibited a higher response, suggesting a correlation between antibody levels and survival.

Notably, the effect of storage temperature variation on vaccine efficacy has also been studied using VSV-LASV in an effort to determine whether strict cold chain conditions are required [115]. Stocks of VSV-LASV were maintained for one week at 4 °C, ~21 °C (room temperature), or 32 °C; an additional stock was subjected to three freeze–thaw cycles over the course of the same week. Hartley guinea pigs were immunized with the sub-optimally stored vaccines and then challenged with guinea pig-adapted LASV 28 days later. Remarkably, all vaccinated animals showed some degree of protection, with the vaccine stored at 32 °C performing the worst but still protecting 66% of the animals. Similar experiments performed with VSV-EBOV showed even better performance, with all vaccinated animals surviving challenge, regardless of the sub-optimal storage conditions. Thus, prolonged and multiple breaks in the cold chain of VSV-LASV and VSV-EBOV do not significantly alter their protective efficacy.

### 3.4. Henipaviruses

Nipah virus (NiV) and Hendra virus (HeV), which both belong to the family *Paramyxoviridae*, are high-consequence pathogens that can cause severe pulmonary and neurologic disease in humans. The VSV platform has been used to develop candidate vaccines against both viruses, although much more work has been done with NiV.

NiV vaccine candidates currently use NiV fusion (F) and attachment (G) glycoproteins as antigens. The rVSV NiV vaccines have been designed according to three strategies: (1) replacement of VSV G with NiV G or F [53]; (2) insertion of NiV G or F into the wild type VSV genome [53]; or (3) replacement of VSV G with EBOV GP and insertion of NiV G or F [51]. Notably, the first strategy results in a replication-incompetent vaccine vector, while the latter two strategies produce replication-competent vectors. Whether the rVSV vector contained VSV G or not, vaccines expressing NiV G or NiV F both elicited neutralizing antibodies in intranasally immunized mice, as did an immunization strategy that combined VSV G-deficient vectors expressing either G or F [53]. Importantly, however, intranasal inoculation of two-week-old mice with the same combination of VSVs expressing G and F resulted in significant mortality [116], raising safety concerns about immunization strategies that involve both the NiV glycoproteins. Nevertheless, immunization of ferrets or Syrian hamsters with these replication-incompetent vaccines provided complete protection against subsequent NiV challenge, demonstrating the efficacy of the individual vaccine vectors [117,118]. In order to develop a safe and effective replication-competent VSV-based NiV vaccine, Debuysscher et al. leveraged the VSV-EBOV platform to generate VSV G-deficient vaccines expressing EBOV GP and either NiV F or G, referred to as VSV-EBOV-NiV F and VSV-EBOV-NiV G, respectively [51]. Both vaccines offered full protection in hamsters against lethal NiV infection when administered 28 days prior to challenge [51]. Incidentally, a similar vaccine expressing the NiV nucleoprotein (N), VSV-EBOV-NiV N, provided no protection [51]. In a follow up study, complete protection in hamsters was achieved following vaccination with VSV-EBOV-NiV G as late as one day before challenge; however, the survival rate dropped to 17% when the vaccine was administered one day after challenge and no protection was conferred when the vaccine was administered three days after challenge [119]. Importantly, African green monkeys immunized with the replication-competent VSV-EBOV-NiV G or the replication-incompetent VSVs expressing F or G were all completely protected from lethal NiV infection, demonstrating the efficacy of each vaccine platform in a “gold-standard” nonhuman primate model [120,121].

A VSV-based HeV vaccine was generated by inserting the HeV glycoprotein (G) gene into the VSV genome downstream of VSV G, such that resulting replication-competent rVSV expressed both glycoproteins [122]. Immunization of BALB/c mice resulted in a robust humoral immune response with high levels of HeV G-specific antibodies and neutralizing activity detected. Further work is required to assess the efficacy of this vaccine in a suitable animal model.

### 3.5. Coronaviruses

VSV-based vaccines have also been developed for diseases caused by the human coronaviruses: severe acute respiratory syndrome coronavirus (SARS-CoV), Middle East respiratory syndrome (MERS)-CoV, and SARS-CoV-2. A replication-competent rVSV expressing the Spike (S) protein of SARS-CoV in addition to VSV G induced neutralizing antibodies in mice after a single vaccination and was able to protect against SARS-CoV challenge 1 or 4 months later [123]. Interestingly, a replication-incompetent rVSV expressing SARS-CoV S instead of VSV G elicited a much more robust neutralizing response in mice, demonstrating the effectiveness of this platform [124]. Liu et al. demonstrated that a replication-competent rVSV expressing MERS-CoV S in place of VSV G was able to induce neutralizing antibodies and T cell responses in rhesus macaques after a single intramuscular or intranasal immunization dose, indicating a potential to confer protection against MERS-CoV infection [125]. Several labs have also developed different rVSV-based SARS-CoV-2 vaccines expressing the S protein of this virus. Each of these VSV-based vaccines has induced neutralizing antibody responses, reduced virus infection, and conferred protection against SARS-CoV-2 infection in mice, hamsters, and nonhuman primates [126,127,128,129]. In particular, a replication-competent rVSV expressing SARS-CoV-2 S instead of VSV G rapidly and potently induced neutralizing antibodies after a single-dose vaccination of hamsters and protected the animals against SARS-CoV-2 challenge [128]. The development of this vaccine has progressed into phase 1/2 clinical trials (https://clinicaltrials.gov/ct2/show/NCT04608305, accessed on 29 July 2021).

### 3.6. Zika Virus

The VSV platform has been used to generate a number of different vaccines against Zika virus (ZIKV), a re-emerging flavivirus. By inserting the ZIKV pre-membrane and envelope proteins (prME) or the pre-membrane and soluble envelope proteins (prMsolE) into the VSV-EBOV backbone, Emanuel et al. generated two novel VSV-based vaccines that capitalized on the immune cell-targeting nature of EBOV GP while still providing ZIKV-specific epitopes [130]. Both vaccines, known as VSV-ZIKVprME and VSV-ZIKVprMsolE, conferred 100% protection against a lethal dose of ZIKV in IFNAR^-/-^ mice when a dose of 1 × 10^4^ PFU was administered intramuscularly 28 days prior to challenge. Similar to VSV-EBOV, VSV-ZIKVprME provided rapid immunity, with 50% of IFNAR^-/-^ mice surviving if immunized one day prior to ZIKV infection and 100% of mice surviving if immunized three or more days prior to infection. Notably, mice immunized with these vaccines were also protected from mouse-adapted EBOV infection. Not only does this study demonstrate the effectiveness of a novel ZIKV vaccine, but it also highlights the potential utility of VSV-EBOV as a platform for designing vaccines against other viruses.

A separate group developed a set of ZIKV vaccines using an rVSV backbone that had been attenuated by abolishing the methyltransferase activity of L rather than removing VSV G [131]. Methyltransferase defective (mtd) rVSVs expressing either the ZIKV pre-membrane protein (mtdVSV-ZIKVprM) or the pre-membrane protein, in addition to the envelope and non-structural 1 (NS1) proteins (mtdVSV-ZIKVprM-E-NS1), were able to elicit potent humoral and cellular immune responses and protect BALB/c and A129 mice from disease associated with ZIKV challenge. Interestingly, a vaccine expressing only NS1, mtdVSV-ZIKVNS1, was able to partially protect A129 and IFNAR^-/-^ mice from ZIKV challenge, likely owing to a robust T cell response since a neutralizing antibody response was absent [131,132].

### 3.7. Crimean-Congo Hemorrhagic Fever Virus

The VSV platform has recently been leveraged to develop a novel vaccine against Crimean-Congo Hemorrhagic Fever Virus (CCHFV), which is a tick-borne bunyavirus that can cause severe hemorrhagic disease in humans. Rodriguez et al. generated an rVSV in which the CCHFV GPC replaced VSV G; however, this virus could only be rescued if VSV G was provided in trans [133]. To circumvent this constraint, the virus was serially passaged first in BHK cells expressing VSV G and then in BHK cells alone. The resulting virus, referred to here as VSV-CCHFV-GPC, contained several mutations in the GPC that rendered it replication-competent. Immunization of STAT-1 knockout mice with 10^7^ PFU of VSV-CCHFV-GPC followed 35 days later by challenge with a lethal dose of 50 PFU CCHFV resulted in complete protection. Administration of a booster dose 14 days after the prime dose also resulted in complete protection, in addition to a reduction in the severity of weight loss.

## 4. Conclusions

From the isolation of VSIV in 1925 to the clinical approval of VSV-EBOV in 2019, VSV’s ascent from agricultural pathogen to model virus and, eventually, proven countermeasure has been remarkable. VSV has played a central role in our understanding of single-stranded, negative-sense RNA viruses, and the molecular tools—including reverse genetics—that have been developed to work with this virus have since been adapted and applied to other viruses. Recombinant technologies created the possibility to use VSV as a surrogate for studying the glycoproteins of high-consequence pathogens, which ultimately resulted in one of the first clinically approved EBOV vaccines and sparked the development of numerous other vaccines. Moreover, although not discussed here, VSV is also being actively pursued as an oncolytic vector, owing to its lytic replication cycle and sensitivity to type I interferon-dependent immune responses [134]. Indeed, VSV is a versatile, safe, and effective platform that has already delivered impactful new countermeasures, with the promise of more to come.

## Data Availability

Not applicable.
